# Quasi-passive lower limbs exosuit: an in-depth assessment of fatigue, kinematic and muscular patterns while comparing assistive strategies on an expert subject’s gait analysis

**DOI:** 10.3389/fnbot.2023.1127694

**Published:** 2023-05-11

**Authors:** Christian Di Natali, Jesus Ortiz, Darwin G. Caldwell

**Affiliations:** Laboratory XoLab, Department of Advanced Robotics (ADVR), Istituto Italiano di Tecnologia (IIT), Genova, Italy

**Keywords:** exoskeleton, soft robot applications, assistive device, wearable robots: exoskeletons and exosuits, human-robot interaction, biologically inspired robotics, biological control systems

## Abstract

Wearable robots are becoming a valuable solution that helps injured, and elderly people regain mobility and improve clinical outcomes by speeding up the rehabilitation process. The XoSoft exosuit identified several benefits, including improvement of assistance, usability, and acceptance with a soft, modular, bio-mimetic, and quasi-passive exoskeleton. This study compares two assistive configurations: (i) a bilateral hip flexion (HA, hips-assistance) and (ii) a bilateral hip flexion combined with ankle plantarflexion (HAA, hips-ankles-assistance) with the main goal of evaluating compensatory actions and synergetic effects generated by the human- exoskeleton interaction. A complete description of this complex interaction scenario with this actuated exosuit is evaluated during a treadmill walking task, using several indices to quantify the human-robot interaction in terms of muscular activation and fatigue, metabolic expenditure, and kinematic motion patterns. Evidence shows that the HAA biomimetic controller is synergetic with the musculature and performs better concerning the other control strategy. The experimentation demonstrated a metabolic expenditure reduction of 8% of Metabolic Equivalent of Task (MET), effective assistance of the muscular activation of 12.5%, a decrease of the muscular fatigue of 0.6% of the mean frequency, and a significant reduction of the compensatory actions, as discussed in this work. Compensatory effects are present in both assistive configurations, but the HAA modality provides a 47% reduction of compensatory effects when considering muscle activation.

## 1. Introduction

The world’s elderly population is expected to increase significantly by 2,050 (estimated to rise to almost 22% of the worldwide population), reaching about 2 billion and doubling the proportion from 2002 (World Health Organization [WHO]., 2015). Against such a background, a major concern that is attracting growing attention concerning the quality of elderly life is the link with physical activities (Dunn et al., 1998). Indeed, when physical activity is reduced, there is a strong correlation with psychosocial problems such as social isolation, unhappiness, or depression. At the same time, a lack of physical activities leads to further muscle degeneration in the lower limbs, with elderly persons potentially falling into a negative cycle of depression, skeletal muscle decline and further decreases in physical activity (Rejeski and Mihalko, 2001).

Mobility is a key component of health throughout all phases of the lifespan, and it is vital to ensuring that older adults can maintain independent functioning and autonomy (Richardson et al., 2015). Mobility limitations, defined as difficulty walking a one-quarter mile or climbing one fight of stairs, are reported by 30–40% of adults aged 65 years and older (Shumway-Cook et al., 2005). Because of these walking difficulties caused by age-related skeletal muscle decline of the lower limbs, many elderly persons partake in fewer and shorter physical activities than young people (Rosenbloom, 1988). One of the main causes of muscle degeneration is that many older adults do not engage in regular walking activities (Colley et al., 2011). Relatively slow gait speed is always detected in the elderly. This is an adaptive response to conserve energy (Alexander et al., 2010). As peak aerobic capacity (VO_2_ peak) declines with increasing age (Waters et al., 1983), the energetic requirements of walking at a given speed increase relative to VO_2_ peak, such that normal walking becomes more intense. Indeed, evidence suggests that energy requirements during walking play a central role in the development of mobility limitation in older adults (Fiser et al., 2010; Schrack et al., 2010).

It is critical to remain active and mobile to slow down the degrading of overall physical health and cognitive functions (Volkers et al., 2012). Indeed many elderly people, due to neuromotor deficits, make use of assistive devices such as canes, walkers, and orthoses to enable walking at home (Jutai et al., 2007). However, many of these devices substitute or complement the functional loss but do not encourage the activation or rehabilitation of the legs. Robotic research is actively trying to address some of the most pertinent problems of an aging society.

Wearable robots may be a solution that helps elderly people to regain their mobility. Recent years have seen the development of powered exoskeletons designed to restore walking in individuals who are unable to walk (Dollar and Herr, 2008; Goldfarb et al., 2013)). The main characteristic of these exoskeletons is the rigid structure that can support their weight and provide high levels of assistance to the wearer. While these devices are actively being studied as an alternative to wheelchairs for paralyzed people, they are too complex and expensive for users with a low to moderate degree of impairment (Ortiz et al., 2021). For the individuals who need some, but less, assistance, a new generation of exoskeletons based on soft technologies is in development, which has excellent promise in terms of usability and performance. They specifically target users that retain some degree of mobility, and consequently, the exoskeleton only provides partial assistance/support. This is well suited to current soft technologies. For example, the Harvard soft exosuit (Awad et al., 2017), or MyoSuit (Haufe et al., 2019), share common elements, such as cable-driven actuation, although they use different approaches in their implementation. The presented trend underlines the need in several applications and target users, where wearability and acceptability become critical features to drive the shift from heavy rigid exoskeletons to light, soft wearable devices. In the XoSoft project, we introduced Quasi-Passive Actuations (QPA) to create a biomimetic device. These QPAs are composed of a Textile-Based Clutch (TBC) (Sadeghi et al., 2019) together with an Elastic Tendon (ET) that forms the passive mechanical element, which is connected in series. From an assessment point of view, the analysis of a passive device is more complex because of the continuous energy exchange between the user and the ETs.

Most of the scientific efforts are focused on conducting kinematic, dynamic and metabolic assessments (Wehner et al., 2013; Shamaei et al., 2014; Di Natali et al., 2019; Zhou et al., 2020), rather than carrying out analysis on the muscle activity (Van Dijk et al., 2011). Inconclusive answers are reported in this (Van Dijk et al., 2011), mainly because of the complexity of the experimental protocol that has been carried out, e.g., multiple subjects, high variability in gender, age and physical characteristics, and the effects of the learning curve in the use of new technological devices. That protocol also has to cope with the high sensitivity and variability of the measurement equipment used (surface EMG for muscular activation measurement). This technology deserves a more accurate analysis to underpin the effective and potential results that this equipment could bring. Thus, a systematic comparative analysis of similar technology (i.e., passive or quasi-passive actuated exosuit) is relevant to better understanding what happens at the muscular level.

## 2. Background and motivation

The rationale behind the assistive approach is based on the typical muscular pattern during walking. During walking, the main muscles activated are the gluteus, gastrocnemius and rectus femoris, acting during stance, push-off and swing phases, respectively, (Winter, 2009). Typical gait assistive strategies proposed in rehabilitation exoskeletons that aim to reduce fatigue during walking, support alternatively hip flexion (Jin et al., 2016), hip extension (Asbeck et al., 2015), or ankle plantarflexion (Collins et al., 2015). Hybrid approaches (Asbeck et al., 2013; Ding et al., 2014) also report good effectiveness in reducing muscular activation or metabolic consumption between 5 and 15%.

**FIGURE 1:**
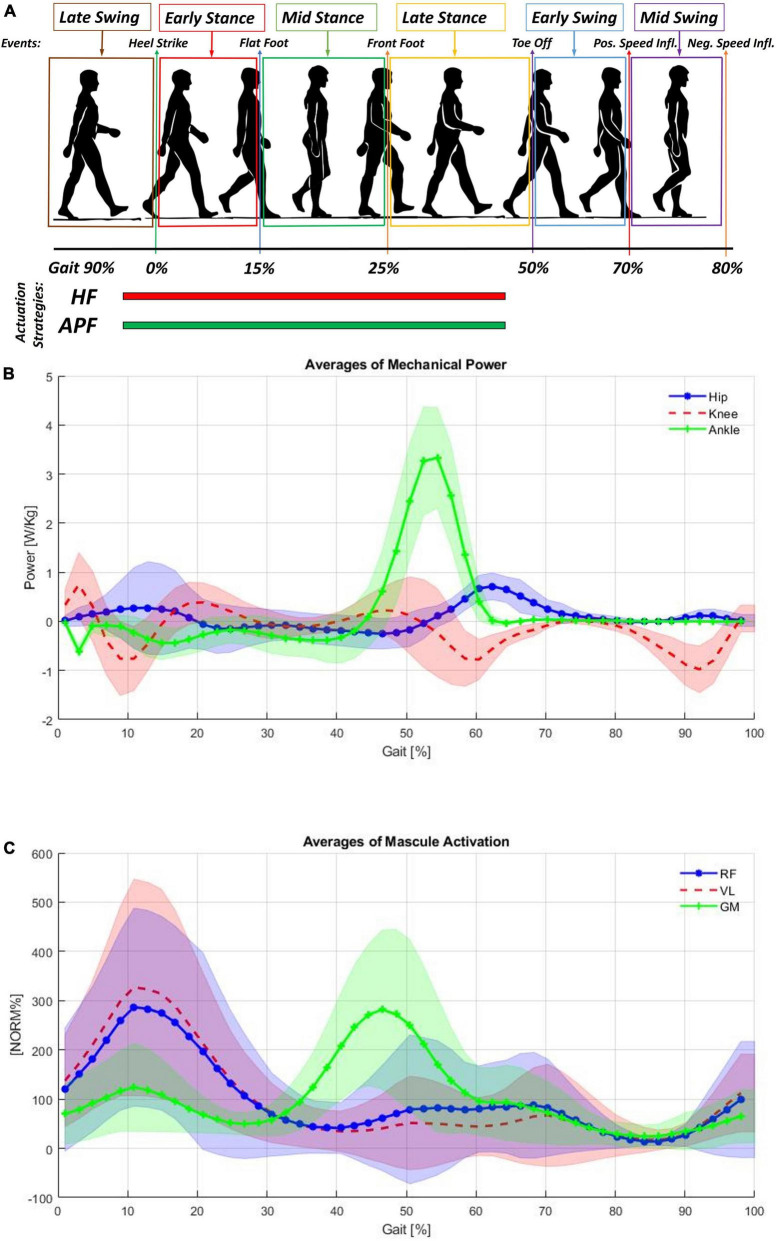
**(A)** Cyclical gait representation, **(B)** averages of mechanical power measured at the hips, knees and ankles normalized for the subject weight (Winter, 2009). **(C)** Shows the averages of muscular activation of the rectus femoris (RF), vastus lateralis (VL), and gastrocnemius medialis (GM). Each subject’s mean EMG was normalized to 100% prior to averaging (Winter, 2009).

During normal walking, as shown in Figure 1B, power is expended by the body primarily at the transitions of support from one leg to the other. This power is mainly provided by the hip (represented in blue) and ankle (represented in green) (Winter, 2009). During stance, the gastrocnemius contracts isometrically, stretching the Achilles tendon due to the body’s natural motion falling forward. Subsequently, these muscles contract concentrically, and the Achilles tendon recoils, giving a large positive power burst from 40 to 60% of the gait cycle to propel the body upward and forward (shown in Figure 1C with the activation of the gastrocnemius medialis). The muscles at the front of the thigh (such as the rectus femoris, shown in Figure 1C) provide a smaller power burst at the hip and a period of power absorption during the transition between legs. The power absorption occurs from 35 to 50% of the gait cycle. The power burst occurs from 50 to 75% of the gait cycle, providing energy to help swing the leg. It is reported (DeVita and Hortobagyi, 2000; McGibbon, 2003) that elderly persons if compared with young persons, are characterized by reduced power generation at the ankle plantar flexors compensated by more power at the hip flexors to guarantee step length. By assisting the hip flexors, the burden on the hip joint would be reduced, and the lower limbs would be lifted sufficiently high that ground clearance of the feet is achieved, and tripping could be prevented (Sposito et al., 2018; Di Natali et al., 2019).

Important aspects, which are also correlated to passive exoskeletons performance (Rahman et al., 2006), are evaluating the effects on gait for the kinematic patterns and muscular activity. Before receiving assistive forces, passive exoskeletons require the user to input energy into the system to elongate the elastic elements. The storing of potential energy causes an increase in muscle activity (Fanti et al., 2022). Moreover, it could also generate changes in motion patterns. Thus, evaluating these changes is critical to understanding the actual efficacy of this complex human-exoskeleton interaction.

In addition, by investigating how the musculature of a healthy subject responds to the assistive and resistive cycles provided by the QPA-based exosuit (Di Natali et al., 2020), we aim to define the technology’s potential for improving mobility and enabling personalized, effective rehabilitation. Consequently, the system acts also as continuous muscle training (Di Natali et al., 2021), which is, in turn, a secondary benefit of the system to help regain mobility by improving gait and postural patterns (Di Natali et al., 2019). Ultimately, considering the complexity of the human musculoskeletal system and the assistance provided, compensatory and synergetic effects on the musculature were studied.

## 3. Objectives and hypotheses

The primary objective of this foundational study is to assess the effective reduction in walking energy consumption and hence quantify the energy exchanges in healthy individuals by using the XoSoft exosuit technology employing QPAs. Moreover, changes in the motion patterns are not evitable. Thus, a synergetic analysis of kinematic and muscular patterns is critical to understand the overall effectiveness of the interaction with the exosuit. Two different assistive modalities have been assessed: (i) bilateral hip flexion (HF) assistance; and (ii) bilateral HF assistance and bilateral ankle plantarflexion (APF) assistance. These two assistance strategies are those most commonly adopted in literature to reduce the energy burden during walking with active exosuits (Asbeck et al., 2013; Ding et al., 2014; Jin et al., 2016; Awad et al., 2017; Haufe et al., 2019). The hypothesis of this study is a comprehensive evaluation of human-exoskeleton interaction focusing on several different indexes that provide positive and negative effects on the system energy exchange and motion patterns during walking activities, which is particularly important for elderly people. The target evaluation will focus on assessing an effective reduction in metabolic expenditure, a decrease in muscular fatigue, and a minimal change of kinematic patterns while reducing muscle activation.

This study is conducted on a single healthy subject with several hours of experience using a quasi-passive exosuit. Exoskeletons perform differently as a function of experience, fitting, particular motion patterns and many other aspects necessary for optimization to improve cross-subject effectiveness (Zhang et al., 2017). Therefore, a personalized assistive approach must be considered a standard approach. Moreover, a generalization of the effectiveness over a small number of subjects in a single testing run is always difficult to be reliable (Van Dijk et al., 2011). This aspect is essential, particularly when considering the effects of the learning curve on the user’s motion style and neuro-motor coordination, particularly when considering changes in the walking pattern and due to the mutual adaptation in which robot and human effectively cooperate and exchange forces (Moreno et al., 2009; Afzal et al., 2020).

Moreover, this work (Theurel and Desbrosses, 2019) underlines the need for more evidence on the impact of assistive forces on neuromuscular coordination and joint kinematics. Therefore, this study has performed long walking tests on an expert exoskeleton wearer and repeated them over multiple days. We aim to measure the effectiveness of the wearer’s interaction with the exosuit without bias due to the bad fitting and learning curve effects. In addition, multiple test repetitions will be essential to apply statistical analysis to the gathered data while investigating the exoskeleton interaction at the muscle activity level over several gaits (Galle et al., 2017).

This work’s main contribution lays on the hypothesis that the assistance targeted on the hip flexion and ankle assistance would promote a more comprehensive and bio-inspired propulsive force from the muscles activated during walking and, simultaneously, reduce the energetic burden associated with locomotion. This aspect is not obvious, particularly when considering passive (or quasi-passive) exoskeletons interacting with such a complex as the human system. In principle, The XoSoft exosuit generates forces on the body during both storing (elastic elongation) and releasing phases, thus mimicking the behavior of muscles and tendons. These forces generate assistance during the realigning phase. The elastic tendon has already accumulated potential energy and releases it to the user.

In contrast, the storing phase is characterized by storing energy from the elastic tendon the user provides. This phenomenon of the wearer’s interaction with a passive actuated exoskeleton is fundamental when considering the overall exchange of forces and energy balance. Moreover, this study also impacts the understanding the fundamental interaction with passive exoskeletons.

## 4. Materials and methods

### 4.1. XoSoft platform

The XoSoft EU project developed a user-centered design-based exosuit. This soft, modular, bio-mimetic and quasi-passive exoskeleton assists users such as the elderly, and post-stroke and partial spinal cord injury subjects with low to moderate mobility impairments (Di Natali et al., 2019). The XoSoft-Gamma prototype demonstrated in Di Natali et al. (2020), features reconfigurable and modular soft pneumatic QPAs to deliver bilateral gait assistance for hip and knee flexion and extension and ankle plantar and dorsiflexion. QPAs relying on variable stiffness mechanical elements, the TBC (Sadeghi et al., 2019), are used to modulate the forces generated by the passive elements employed to store the mechanical energy.

The XoSoft exosuit was designed to support the user at the hips and ankles in this work. The ETs characteristics and possible control strategies are extensively presented in Di Natali et al. (2020). The ETs selected for this study are reported in Table 1. The exosuit is designed to assist power absorption through controlled modulation of the extension of the passive element. The exosuit is actuated for both hip flexion (HF) and ankle plantarflexion (APF) actuation from 5 to 65% of the gait cycle (the timing of both control strategies are shown in Figure 1A, with the hip flexion presented in red, and the ankle plantarflexion in green).

**TABLE 1:** Characteristics of the et used in experimental trials.

ET for specific joint	Stiffness (N/mm)	ET length (mm)	Force at 50% of ET elongation (N)
Hip flexion (HF)	1.6	50	40
Ankle plantarflexion (APF)	2.5	20	25

**FIGURE 2:**
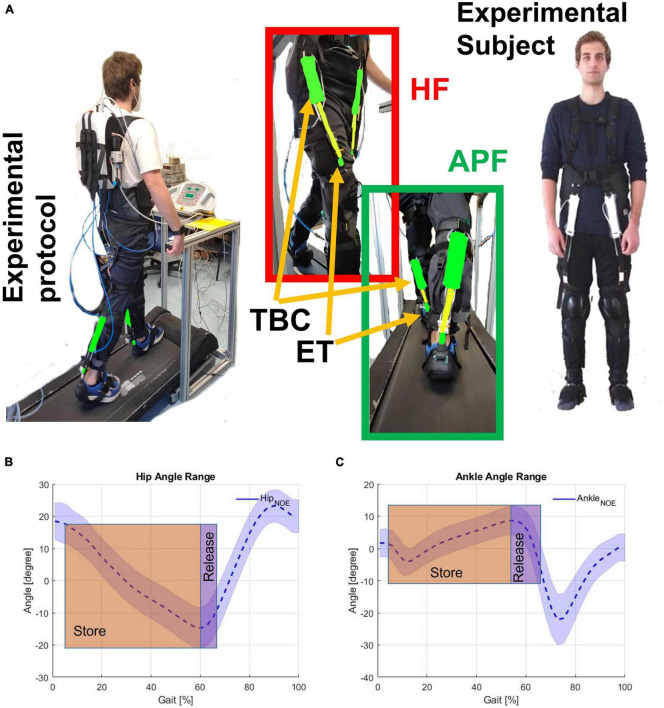
**(A)** Experimental subject wearing the exosuit. Hip flexion (HF) and ankle plantar flection (APF) are shown together with the textile-based clutch (TBC) and the elastic tendon (ET). Store and release phases on the **(B)** HF (hip angle), and **(C)** APF (ankle angle). The human subject gave permission for the use of their image.

The wearer, thus, before receiving assistance, has to exert a force to elongate the ETs in an energy-storing phase. Subsequently, the exosuit returns this stored energy to the wearer in the releasing phase (see Figures 2B, C). If the system efficiency were ideal at the end of the cycle, the physical energy balance would be the same. Still, previous works demonstrated that control strategies could modify this balance toward a surplus value of assistance (Di Natali et al., 2019, 2020, 2021).

Figure 2A shows the subject wearing the XoSoft exosuit configured with the assistive modules, i.e., QPA hip flexion and QPA ankle plantarflexion to assist at the hip and ankle, respectively. The effective elongation and the torque generated by each actuator are a function of the corresponding articulation angle shown in Figures 2B, C. Walking is a cyclical motion characterized by oscillating trends of the lower limbs. This determines the gait cycle portions to be exploited for the elongation of the ET (storing phase) and the subsequent releasing phase (the effective assistance). We hypothesized that the extraction and storage of mechanical energy during stance would have less impact than its release during the swing, thus shifting the energy balance toward a net assistive condition. The specific HA (hips assistance) strategy engages the QPA between 5 and 65% of the gait cycle, where the elastic energy is accumulated during the stance and then returned during the swing. The HAA (Hips-ankles-assistance) strategy also assists the gastrocnemius between 5 and 65% of the gait cycle to propel the body upward.

Similarly, the QPA requires a storing phase before releasing assistance to the target ankle. During stance, the QPA ankle plantarflexion is engaged. Consequently, the ET stretches thanks to the body’s motion “falling” forward. Subsequently, when the person starts pushing, and the ankle angle reaches its minimum, the user actively contracts the calf muscles. The ET contracts to its original length, releasing the stored mechanical energy and aiding the propulsion of the body upward. Figures 2B, C show the concept underpinning both the storing and releasing phases of the HA and HAA strategies as functions of the corresponding joints.

### 4.2. Design of the study

The experimentation focused on evaluating, during treadmill walking, the effects of the QPA-based exosuit on healthy gait mechanics and energetics. The assessment of the reduction in walking energy requirements was evaluated in three experimental configurations (i) the baseline (NOE), (ii) the HA and (iii) the HAA configurations.

This study aims to meticulously detail the effects of using a QPA-based exosuit on muscle patterns, muscle synergy, and compensatory effects. Experimentation is conducted on a single healthy subject with experience in using XoSoft to reduce subject-specific performance variability, which was involved in completing each test repetition with and without the exoskeleton worn. The test is repeated five times on different days applying for the following order between the three conditions: NOE, HA, and HAA. Moreover, the test was performed five times on different days to reduce set-up variability due to exosuit wearing and sensor placement. In addition, we imposed a rest period of 20 min between each repetition, during which the subject recovered, and the investigators controlled the equipment. Single-subject experimentation is critical, particularly when measuring errors introduced by intra-subject variability and subject-specific performance variability. The main objective is to analyze muscular and kinematic pattern changes and compensatory effects while reducing any possible risk of affecting measurements due to the errors introduced by the experimental protocol, e.g., multiple measurements, donning-doffing wearable devices, dynamic movements, and sweating.

A 5-day testing protocol was used to evaluate the effects of the two different exosuit assistive configurations (HA and HAA). During any single testing day, the three different walking tests were performed for 10 min on a treadmill at a natural self-selected walking speed of 3 km/h (0.83 m/s), taking approximately 600 steps for each leg. Data were recorded for the monitored muscles, namely: Rectus Femoris (RF), Vastus Medialis (VM), Tibialis Anterior (TA), and Gastrocnemius Lateralis (GL). Apart from the gluteus, these four selected muscles are the most relevant for gait analysis. The gluteus was not selected due to possible interference with the exosuit. The Biceps Femoris or the hamstrings are also relevant in the walking pattern. In this work, the hamstrings were not monitored since the main assistive effects of the exosuit of the hips motion would mainly exert on the hip flexors. Moreover, our previous work (Fanti et al., 2022) reports results on a similar exosuit assistive configuration on the semimembranosus, which is responsible of hip extension. To apply a statistical approach, right and left muscle activations were averaged for each stride, determining the overall behavior for each studied configuration and analyzed muscles. Each test modality was compared against the specific daily baseline (NOE) to minimize the day-to-day variability of the participant.

### 4.3. Experimental protocol

This work aims to evaluate if specific assistive exosuit control profiles reduce metabolic energy and muscle fatigue during walking at a fixed rate. Biomechanics and energetic considerations are reported during treadmill walking on three experimental conditions: (i) the reference condition without the exoskeleton being worn (NOE), (ii) the hip flexion assistance study referred to as HA, and (iii) the hip flexion and ankle plantarflexion assistance study referred to as HAA. Two primary outcomes were assessed: the activity of four muscles (RF, VM, TA, and GL), and the energy cost of walking, defined as mass normalized oxygen consumption (ml O_2_/kg). From a kinematic point of view, maximum extension and flexion are evaluated, along with the range of motion (RoM) of the hip, knee, and ankle. All trends are plotted within each gait cycle and are expressed as a percentage of the stride period. 0% refers to the heel strike, and 100% to the next consecutive heel strike. All results are reported by combining right and left signals over a 10-min walking test, repeated five times for each test scenario, thus averaging approximately three thousand strides. Muscle activations were normalized over the Maximal Voluntary Exertions (MVE) computed as the 95th percentile of the baseline distribution of the four EMG measurements taken during normal walking at 3 km/h. This approach prevents the false selection of maximum muscle activations due to erroneous EMG spikes. The muscle activations vary from 0 to 1 of MVE (where 1 corresponds to 100% of MVE of each muscle during walking) in all four measured muscles.

The experiment was approved by the Ethical Committee of Liguria (protocol reference number: CER Liguria 001/2019) and complied with the Helsinki Declaration. A healthy adult participated in the study (male, age 35 years, height 1.70 m, weight 70 kg). After fully explaining the experimental procedure, the subject signed a consent form before participating. We recorded the participant’s fully-body kinematics, lower limb surface electromyography (EMG) and metabolic expenditure during the test. An Xsens wearable motion tracking system was used to record full-body kinematics (MTw Awinda 3D Wireless Motion Tracker, Xsens Technologies B.V. Enschede, Netherlands) at a sampling rate of 100 Hz. An 8-channel Wi-Fi transmission surface electromyography (FreeEMG 300 System, BTS, Milan, Italy) was used to acquire the surface myoelectric signals (sEMG) at a sampling rate of 1,000 Hz. K5 metabolic wearable technology (K5 COSMED Srl, Roma, ITALIA) was used to measure the metabolic expenditure. After skin preparation, bipolar Ag/AgCl surface electrodes (diameter 2 cm) prepared with electro-conductive gel were placed over the muscle belly of RF, VM, TA, and GL in the direction of the muscle fibers (distance of 2 cm between the center of the electrodes) according to the European recommendation for surface electromyography (Hermens et al., 2000) and the atlas of muscle innervation zones (Barbero et al., 2012). The baseline muscle activity and energy expenditure were recorded without the exoskeleton. Each repetition was conducted on a treadmill, with the speed set to the participant’s natural and self-selected overground walking speed (approximately 3 km/h).

### 4.4. Data analysis

Data were processed using MATLAB software (MATLAB 2020, MathWorks, Natick, MA, USA). The raw EMG signals were band-pass filtered using a zero-lag third-order Butterworth filter (20−450 Hz), rectified, and low-pass filtered with a zero-lag fourth-order Butterworth filter (10 Hz). The time scale was normalized by interpolating individual gait cycles over 1,200 points. Then, the EMG signal from each muscle was normalized to the MVE value across all trials. The MVE is recorded at the start of each experimental day when the muscles were fresh and then used as the maximal values across the successive measurements. The Results section reports the trends of each muscle activation against the baseline (NOE), particularly highlighting the resistive and assistive phases of the gait (Di Natali et al., 2020). The resistive phase is when the muscle activity is higher than the baseline, while the assistive phase is when muscle activity is lower than the baseline. The specific assistive strategy (HA and HAA) can strongly affect the oscillation of the muscle activity about the baseline value. The Results section extensively studies and reports the phases of resistance and assistance.

The root means square (RMS), and the Mean Frequency (MF) of the power spectrum of the EMG signals were calculated to investigate the effect of the exosuit on muscle fatigue. For each stride, the RMS was computed over specific intervals of the gait cycle for each muscle, according to the following formula:


$$R\,M\,S=\sqrt{\frac{1}{N}\,\sum_{i=1}^{N}E\,M\,G_{i}^{2}}$$

Where EMG_*i*_ is the value of the ith sample of the envelope of each muscle and N is the number of samples of each interval. For each muscle and each stride, the MF was computed as the ratio between the spectral moments of order 1 and 0 (Ament et al., 1993; Dimitrov et al., 2006):


$$M\,F=\frac{\int_{t_{1}}^{t_{2}}f\,P\,S\,D\,\left(f\right)\,\delta \,f}{\int_{t_{1}}^{t_{2}}P\,S\,D\,\left(f\right)\,\delta \,f}$$

Where t_1_ and t_2_ are the initial and final instants of each stride, PSD(f) is the power spectrum density of the EMG signal, and f is the frequency.

The estimation of the metabolic cost is based on an indirect measurement of the oxygen consumption and respiratory quotient as presented in Goedecke et al. (2000); Jin et al. (2016). The mathematical equation used to derive the metabolic energy expenditure (EE) expressed in watts [W], is a function of oxygen consumption (V_*O2*_) and the respiratory exchange ratio (RER) as in:


$$E\,E=c_{1}\,V_{O_{2}}\,\left(c_{2}\,R\,E\,R+c_{3}\right)$$

Where the conversion factors are: c_1_ = 69.7, c_2_ = 1.2341, and c_3_ = 3.8124, the respiratory exchange ratio (RER = V_*CO2*_/V_*O2*_), as reported in Jansson (1982), is the ratio between the CO_2_ produced and the O_2_ used during metabolism.

Joint angles were calculated based on the standards defined by the International Society of Biomechanics (Grood and Suntay, 1983). All kinematic data were plotted from 0 to 100% of the gait cycle, where 0 and 100% are the consecutive touches of the same heel. The measurements of each lower leg joint angle were averaged over each right and left gait segmentation to estimate and display averaged trends.

Both kinematic, muscular, and exoskeleton data are fully synchronized with a common triggering signal. As previously mentioned, the data is then segmented, and the analysis is represented over the gait cycle. The objective is to evaluate the averaged behavior of muscles and joint angles over multiple test days. The reason for multiple testing days is not to propose a longitudinal test but to enlarge the data set without impacting the user’s fatigue due to longer trials. A multiple testing days approach will also provide a big data set that, averaging each signal over the gait cycle, will allow achieving mean behaviors of the target signals over the gait cycle. Thus, sudden errors and irregular motions would be smoothed down while similar motions would be emphasized. The statistical analysis was performed for the MF and the estimation of the metabolic cost data using SPSS 20.0 software (IBM). *P*-values < 0.05 were considered statistically significant. We used the Shapiro–Wilk test (Ghasemi and Zahediasl, 2012) to verify that the data was from a normal distribution. Then we applied a parametric paired *t*-test to detect any significant differences. For the muscle and kinematic representation, a descriptive statistical analysis was applied to evaluate the average behavior and variations (STD) over the 6,000 strides (600 strides for leg for each of the 5 days of test) for each assistive modality (NOE, HA, and HAA). The bold line in the muscles and kinematic trend plots represents the averaged behavior of muscle activities and joint angles. At the same time, the shaded region covers about 68% of the variation of the data around the mean values.

## 5. Results

### 5.1. Metabolic energy consumption results

**FIGURE 3:**
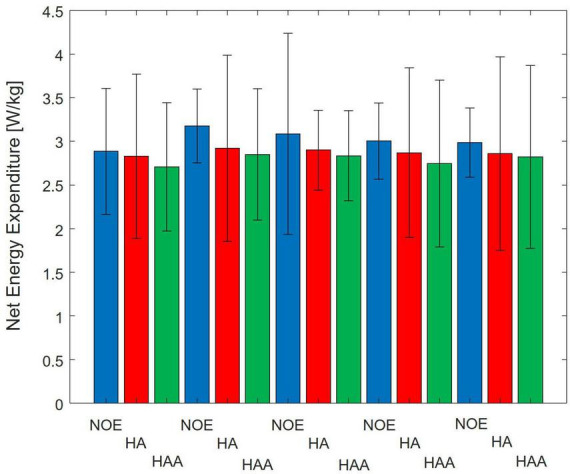
Comparison of Baseline vs. Hips-assistance (HA) and Hips-Ankles-assistance (HAA) modalities of the metabolic consumption trends of all 5-day tests. Numerical results are normalized on the subject mass.

In this section, both assistive strategies (HA and HAA) are assessed against baselines and compared regarding metabolic expenditure variation. Figure 3 reports the energy cost associated with 10 min of walking for the three configurations. The average metabolic expenditure normalized for the subject’s weight of normal walking measured over five tests using the NOE configuration is 3.02 ± 0.62 W/kg, (2.6 Metabolic Equivalent of Task - MET). Where 1 MET is equal to 1.162 W/kg. For the HA configuration, the metabolic expenditure is 2.88 W/kg (2.5 MET), while the HAA configuration reports consumption of 2.79 W/kg (2.4 MET). Table 2 shows the relative reduction and *p*-value of the *t*-test.

**TABLE 2:** Comparison of HA and HAA strategies concerning the baseline (NOE): 5 days average of the relative reduction of metabolic expenditure and *p*-value.

	Relative reduction of metabolic expenditure (%)	*t*-test, *p* < 0.05
HA vs. NOE	4.98	0.0191
HAA vs. NOE	7.75	0.0031

### 5.2. Muscle fatigue analysis results averaged over 5 days: HA and HAA comparison

**FIGURE 4:**
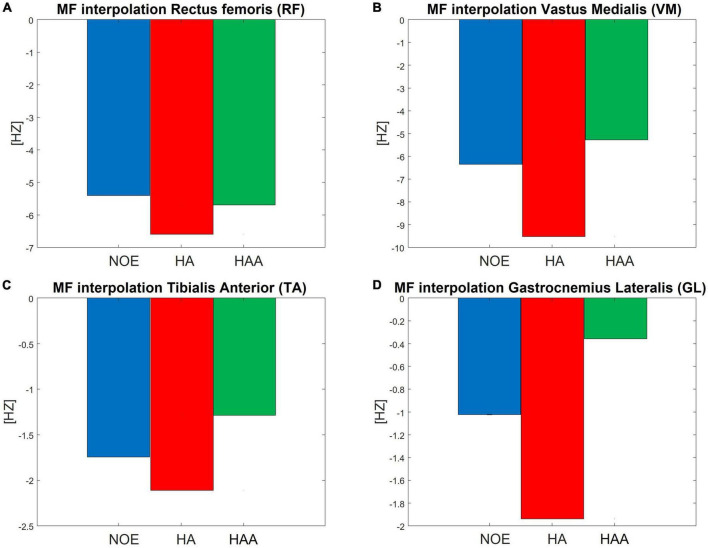
Normalized mean frequency (MF) measured as the difference between the initial and final stride during a 10 min walking test, for the **(A)** RF, **(B)** VM, **(C)** TA, and **(D)** GL.

This analysis shows the main change in the EMG signal in the frequency domain with a spectrum translation toward lower frequencies (Cifrek et al., 2009). Thus, if the specific MF of a single muscle decreases during the test, the user perceives increased fatigue in that muscle. The MF is calculated as shown in the Data analysis section for each stride (18,160 strides in total if considering the right and left leg of the NOE, HA, and HAA configurations). Subsequently, each trend is normalized using the initial value of the baseline. Figure 4 shows the normalized MF of each muscle as the difference between the initial and final stride. The baseline of the MF for all four muscles, measured without the exosuit (NOE), shows negative values, underlining the presence of a certain degree of muscular fatigue that occurs during the task. The MF trend decreases linearly in all muscles and configurations as the number of strides increases. A linear envelope is used to average the whole trend for each MF. The barplot of Figure 4 represents the decrease in the MF over the entire walking test, thus indicating muscular fatigue. The same approach has been taken for exoskeleton configurations and all four measured muscles shown in Figures 4A-D. Focusing on the baseline, the MF of the RF during the walking test decreases by 6.9% on average, corresponding to 5.4 Hz at an initial value of 78.8 Hz. The VM starts at 73.4 Hz and decreases by 6.4 Hz. Thus, the mean decrement in 10 min of walking is 8.7%. The TA starts from 77.8 Hz and decreases by 1.7 Hz, thus the mean reduction during the test is 2.2%. Finally, the GL starts from 85.5 Hz and decreases by 1.0 Hz, giving a mean reduction of 1.2%.

We hypothesize that using an exosuit would lead to a smaller decrease in the MF, which indicates of reduced fatigue. This should occur even though the user has to carry the extra weight of the device. For the HA, the results show an increased negative trend of MF for all four evaluated muscles. The RF decrease is 6.6 Hz, which is 8.4% of the initial baseline value. The VM decrease is 9.5 Hz, which is 13.0% of the initial baseline value. The TA decrease is 2.1 Hz, which is 2.7% of the initial baseline value. Finally, the GL decrease is 1.9 Hz, which is 2.3% of the initial baseline value.

For the HAA, the results show an effective reduction of the negative trend of the MF for three of the four muscles under evaluation. The RF decrease is 5.7 Hz, which is 7.2% of the initial baseline value. The VL decrease is 5.3 Hz, which is 7.2% of the initial baseline value. The TA decrease is 1.3 Hz, which is 1.6% of the initial baseline value. Finally, the GL decrease is 0.4 Hz, which is 0.4% of the initial baseline value.

The average of the relative difference in MF across all four muscles of the HA configuration presented in Table 3, shows an increment of the relative difference of the muscle fatigue of 1.8%. The HAA configuration shows a reduction in fatigue of 0.6%, which is also measured as the average of the relative difference of MF for the HAA.

**TABLE 3:** Comparison on the 5 days average of the relative reduction of MF for the HA and HAA strategies with respect to the baseline (NOE).

	Absolute difference of MF (Hz)	Relative difference of MF (%)
HA vs. NOE @ Rectus femoris (RF)	1.2	1.5
HA vs. NOE @ Vastus medialis (VM)	3.2	4.3
HA vs. NOE @ Tibialis anterior (TA)	0.4	0.5
HA vs. NOE @ Gastrocnemius lateralis (GL)	0.9	1.1
HAA vs. NOE @ Rectus femoris (RF)	0.3	0.4
HAA vs. NOE @ Vastus medialis (VM)	−1.1	−1.5
HAA vs. NOE @ Tibialis anterior (TA)	−0.5	−0.6
HAA vs. NOE @ Gastrocnemius lateralis (GL)	−0.7	−0.8

Positive values correspond to an increase in muscular fatigue, while negative values indicate a reduction in fatigue.

### 5.3. HA and HAA assistance strategies comparison on the kinematic analysis

#### 5.3.1. HA: hips-assistance kinematic analysis

**FIGURE 5:**
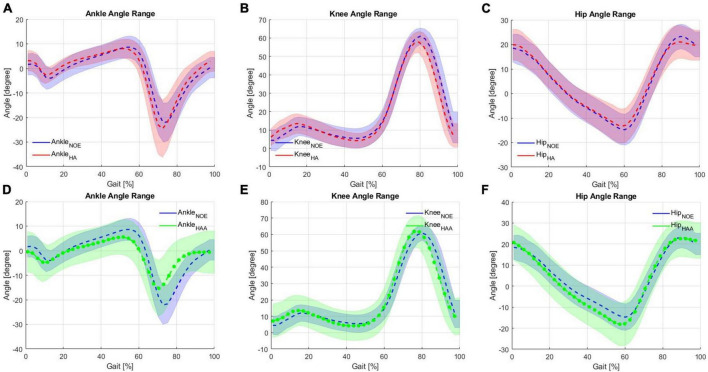
Kinematic analysis comparison on the Hips-assistance (HA) modality for the **(A)** ankle, **(B)** knee and **(C)** hip against baseline during walking on a treadmill, and Hips-Ankles-assistance (HAA) modality for the **(D)** ankle, **(E)** knee and **(F)** hip.

In this section, the HA strategy is assessed against the baseline. The participant produced a physiological range of motions for each of the three lower limb joints (hips, knees, and ankles) while walking on the treadmill at 3 km/h (0.83 m/s). The RoM for the ankle varied from −22° to 9° ± 15° (Figure 5A). For the knee, the range was from 5° to 60° ± 15° (Figure 5B), and for the hip, the RoM was from −15° to 25° ± 15° (Figure 5C). Figures 5A-C shows the joint angle displacements generated using the HA modality. These displacements are averaged over the right and left sides of the body during 10 min of consecutive striding for the ankle, knee, and hip, respectively. Biomechanical consideration of the averaged modification in the behavior of joint angles due to the use of the HA assistive modality shows a slight reduction in both hip flexion (2.24° ± 7.4° between 85 and 100% of the gait cycle) and hip extension (1.4° ± 8.17° from 50 to 70% of the gait cycle) during the late swing and stance phases, respectively, shown in Figure 5C. The exosuit with HA modality reduces the extension by about 3.63° ± 7.85°. This is due to the force generated by the exoskeleton while raising the leg and approaching the swing phase. Consequently, to elongate the ET of the QPA at the beginning of the stance phase, the hip extension is then increased. Hence, the user compensates for this reduction in extension by increasing flexion. The plots for the knee and ankle show similar effects when using the HA modality (shown in Figures 5 A-B). The ankle angle, specifically dorsiflexion, increases from 0 to 30% of the gait cycle by 1.57° ± 5.98°. From 50 to 70% of the gait cycle, plantarflexion is also increased by 2.46° ± 14.33° showing an overall increase in RoM of 1.88° ± 14.26° when compared to the baseline. The knee flexion angle is reduced by about 3.17° ± 6.59° during late swing (70−100% of the gait cycle), while the RoM is also reduced by approximately 2.96° ± 7.4.

These changes in the angular displacements, which are very little (varying from 1.5 to 3.5° approximately of the knees and ankles), underline the fact that, due to the exchange of forces with the exosuit, the walking pattern of the user has been slightly modified. Considering these as compensatory effects, the changes in ROMs are almost negligible compared to natural variations. Moreover, the first external force encountered by the user during the initial part of the gait cycle is in the opposite direction to the user’s motion, which is required to elongate the ET. This force causes the user to compensate slightly for the interference created by the ET by varying the RoM of all three articulations. The ankle shows an increase in the RoM of approximately 6.1%, the knee shows a reduction in RoM of 5.2%, and the hip also indicates a decrease of the RoM of 9.6%. A slight reduction in the RoM of all three articulations is desirable, meaning that the assistance does not significantly affect the natural movement.

#### 5.3.2. HAA: hips-ankles-assistance strategy kinematic analysis

In this section, the HAA strategy is assessed against baselines. Figures 5D-F shows the joint angle displacements compared to the configuration when the exoskeleton is not worn (NOE). Use of the HAA strategy causes decreased dorsiflexion (this effect is visible from 30 to 70% of the gait cycle) of 3.18° ± 9.47° and reduced plantarflexion of 6.73° ± 11.63°, from 70 to 95% of the gait cycle shown in Figure 5D. This behavior shows an overall relative reduction of 32.2% in the RoM and an absolute ankle angle variation of 9.9° ± 13.5° over an average baseline range of 30.75°. Figure 5E shows the knee angle trend. The initial angle during extension and flexion is slightly increased by approximately 2.83° ± 11.64° during the extension phase at 0−20% of the gait cycle and by approximately 0.75° ± 9.27° during the flexion phase (60−80% of the gait cycle). For the hip angle, a slight shift of the hip extension (from 10 to 65% of the gait cycle) generates an increase of 3.4° ± 10.76° shown in Figure 5F. The knee shows a relative reduction in the RoM of 1.86%, and the hip shows a reduction in RoM of 7.3%. These changes in angular displacements, particularly for the ankles, underline a certain degree of unwanted influence and compensatory effects arising from the external forces generated by the exosuit (i.e., forces required to elongate the ETs). This effect is more pronounced on the ankle, where there is an RoM reduction of about 32%, with an average range of 20.8° [residual RoM of 68% concerning the baseline, which is just at the limit to be acceptable before being considered as a pathologic pattern (Bonnefoy and Armand, 2015; Serrao et al., 2017)]. On the other hand, both the hip and the knee trends show a small increase in RoM. This configuration reports a small variation of the RoM, thus not significantly affecting the natural movement.

### 5.4. HA and HAA assistance strategies comparison on the muscular analysis

#### 5.4.1. HA: hips-assistance strategy muscular analysis

**FIGURE 6:**
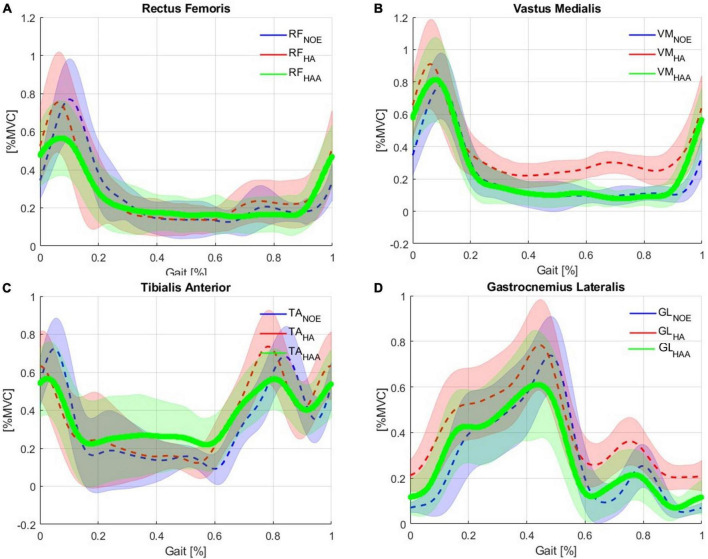
Comparison of Baseline vs. Hips-assistance (HA) and Hips-Ankles-assistance (HAA) modality of the muscle activations of the **(A)** RF, **(B)** VM, **(C)** TA, and **(D)** GL.

Figures 6A-D shows the muscular activation of the RF, VM, TA, and GL, respectively. All the plots are segmented and averaged over each gait cycle. Figure 6 shows the data for the muscle activation averaged over approximately 600 right and left gait cycles for each of the five runs. As expected, the measurements of muscle activation and joint angles are comparable with the normal average behavior of the state-of-the-art available data as in Winter (2009). All baselines are reported in blue in Figure 6. The effects of using this exosuit, with QPA and the HA strategy, are evaluated and compared against the baseline, with the baseline shown in blue and red representing the exosuit with the HA strategy. When the muscle activity of the HA-assisted device is lower than the baseline, this means that only a small amount of muscle activity is required for that specific portion of the gait phase due to the direct assistance provided by the exosuit, or eventual positive synergetic effects from interactions between muscle activations and different walking patterns. More effort is required for that specific gait section with higher muscle activity than the baseline. This effort could be associated with the storing phases (elongation of the ET) or possible indirect compensations arising from changes in the walking pattern. Moreover, the muscular trends show a temporal shift in the muscle activation of about 8% of the gait cycle compared to the baseline. The main numerical results are reported in Table 4.

**TABLE 4:** Muscles assistance and resistance of the HA as comparison with the baseline (NOE).

Muscle	Assistance: portion of gait cycle (%)	Assistance (%MVE)	Resistance: portion of gait cycle (%)	Resistance (%MVE)
Rectus femoris (RF)	10−40	7.6 ± 15.8	60−100, and 0−10	5.2 ± 16.4
Vastus medialis (VM)	10−20	5.9 ± 20.3	20−10	16.3 ± 16.9
Tibialis anterior (TA)	0−15, and 80−90	11.0 ± 18.4	15−80	8.9 ± 13.8
Gastrocnemius lateralis (GL)	50−60	7.1 ± 17.5	60−50	14.1 ± 12.5

#### 5.4.2. HA: hips-ankles-assistance strategy muscular analysis

Figures 6A-D shows the muscular activation of the RF, VM, TA, and GL, respectively. Comparing the HAA strategy against the baseline shows a temporal shift in the muscle activation of about 5% of the gait cycle. The characteristic maximum for each of the four muscles occurs earlier in their respective gait cycles. The main numerical results are reported in Table 5.

**TABLE 5:** Muscles assistance and resistance of the HAA as comparison with the baseline (NOE).

Muscle	Assistance: portion of gait cycle (%)	Assistance (%MVE)	Resistance: portion of gait cycle (%)	Resistance (%MVE)
Rectus femoris (RF)	5−30	7.7 ± 16.5	40−70, and 90−100	4.3 ± 9.8
Vastus medialis (VM)	15−30	2.1 ± 9.1	90−15	6.1 ± 16.9
Tibialis anterior (TA)	0−15, and 70−90	11.3 ± 17.7	20−70	9.4 ± 14.6
Gastrocnemius lateralis (GL)	40−60	9.1 ± 13.7	90−20	4.6 ± 11.1

### 5.5. HA and HAA muscular analysis comparison: relative assistance and resistance

**FIGURE 7:**
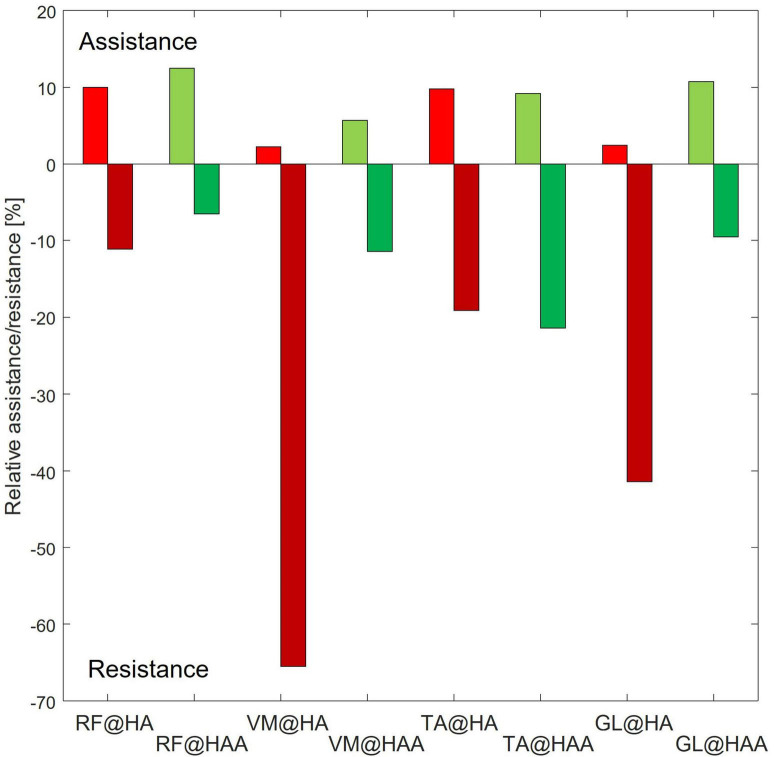
Relative assistance and resistance for each of the assessed muscles [rectus femoris (RF), vastus medialis (VM), tibialis anterior (TA), and gastrocnemius lateralis (GL)] at specific assistive configuration.

To quantify and compare the assistance and resistance effects, shown in Figure 6, the average trends of muscle activity associated with the HA and HAA have been subtracted from the baseline and normalized against the maximum muscle variation across all the tests. Positive and negative relative variations of muscle activities of the HA and HAA strategies for each muscle are separately computed to estimate the relative assistance and resistance. Assistance is when the muscle activity is below the reference baseline, and the resistive phase is when the muscle activity is higher than the baseline. The difference in muscle activity compared to the baseline is averaged and normalized concerning the mean value of the selected baseline section. Then, the result is normalized over the ratio between the time portion of both assistive and resistive phases and the whole averaged gait time. The barplot in Figure 7 represents, with negative values, the muscle activity that the user must provide to store energy in the exosuit (to elongate the ETs).

In contrast, the positive part of the barplot represents the assistance provided by the exosuit and reduces muscle activation. Positive assistance and negative resistance values are generated by comparing the muscle activation of the specific muscle and control strategy against the baseline. The comparison is calculated at the specific interval of the gait cycle, as reported in Tables 4, 5 for HA and HAA, respectively. The percentage of resistance and assistance is derived as a difference between the baseline muscle activity and specific control strategy and then normalized on the baseline value. The system aids 10.0% and a resistance of 11.1% for the RF in the HA configuration. The VM shows assistance of 2.2% against the resistance of 65.5%. The TA shows assistance of 9.8% with a resistance of 19.1%, and the GL shows assistance of 2.4% and a resistance of 41.4%. The system assists 12.5% with a resistance of 6.5% for the RF in the HAA configuration. The VM shows assistance of 5.7% and a resistance of 11.4%. The TA shows assistance of 9.2% against the resistance of 21.4%, and the GL shows assistance of 10.7% against the resistance of 9.5%. When considering the improvement from the HA to the HAA modality, the resistance is reduced by 47%. This reduction is calculated as the difference of each resistance measured at the four muscles for each modality (HA and HAA). Then the differences are normalized over the resistance measured for the HA mode. It is important to underline that even if the energy can neither be created nor destroyed, the energy stored in the ETs generates unbalanced resistive and assistive effects on the muscles. This is closely correlated to the behavior of the human biomechanical system and the fact that humans can react differently during specific gait phases, in some instances extracting or injecting energy (Di Natali et al., 2019, 2020; Fanti et al., 2022).

## 6. Discussion

The functionality of the QPA requires an important interaction between the user and the exosuit. This interaction is characterized by a continuous mechanical energy exchange between the wearer’s biomechanical system and the actuators’ ETs. Due to the high complexity of the human system, it is not too obvious that adding multiple actuators would generate a higher assistive impact. We demonstrated in previous studies that the same exosuit platform could generate assistance by reducing the torque and mechanical power of the wearer (Di Natali et al., 2020). Still, also it could provide just resistance for specific muscle training (Di Natali et al., 2021). Finally, this work (Fanti et al., 2022) shows an oscillation of both muscle activity and mechanical power about the reference signals, thus, underlying complex behaviors of the whole system composed of wearer and exosuit. These results are a function of specific assistance strategies such as actuation placement and control timing. Therefore, in this study, a comprehensive analysis of metabolic cost, muscle fatigue, muscle activity and kinematic patterns has been conducted to provide a clear view of what is happening during the interaction with the exosuit.

The detailed analysis of the performance of the exosuit shows a reduction in the metabolic cost in user fatigue using both exosuit configurations, generating better performance with the HAA. For the muscle fatigue data, we see that the fatigue is strongly reduced for the HAA configuration, but this was not found with the HA setup. The seemingly conflicting results prompted further investigation with more attention on muscle activation and kinematic analysis. The detailed muscle activation analysis showed that the four main muscles involved are sensitive to the actuation configuration. Causing a more (or less) effective net reduction in muscular activity when compared against the baseline, as shown in Figures 6, 7. Thus, both assistive and resistive phases, measured in all four muscles during the gait cycle, are strongly correlated to the selected configuration. This suggests that synergetic and compensatory effects occur even if a specific control strategy at the muscle level reduces metabolic cost. Thus, it is critical to evaluate the interaction between exoskeletons and exosuit, focusing on several indexes as shown in this study. A more comprehensive analysis is necessary to assess particular side effects generated while modifying the complex balance of human motion. Therefore, when considering the full complexity of the compensatory and synergetic effects, results show that assistance applied by an exosuit might partially guarantee advantages. Moreover, the interaction between the assistive device and the whole body must be studied, analyzed, and understood. In addition, a more biomimetic control approach can generate advanced performances with reduced compensatory effects, as shown in this work when using the HAA and HA control strategies.

With this study, we want also to underline the evidence of the compensatory effects, which are shown in Figures 6, 7. This is particularly evident for the VM and the GL, where a more biomimetic controller (the HAA) reduces the compensatory effects. By comparing the HAA and the HA configurations, we noticed an increment of the assistance rather than the resistive effect. This, however, is not valid when considering the TA. Indeed, there is a slight worsening (from the HA to the HAA) of both assistive and resistive effects. The TA suffers if both actuators are applied bilaterally to the hip flexion and the ankle plantarflexion. This actuation strategy generates compensatory behaviors, modifying the standard muscular activation pattern. The development of modified control strategies that seek to address this effect will form part of future work in this area.

### 6.1. Discussion on the metabolic energy consumption

The principal objective was to evaluate the effective reduction of metabolic expenditure in both the HA and HAA exosuit configurations. The results, reported in Table 2, show a net reduction of metabolic expenditure for both configurations concerning the baseline (NOE). Despite the added weight of the device and the interaction with the exosuit, the HA reduces 5% of the total metabolic cost, while the HAA reduces about 8%. The HAA configuration strategy performs 1.55 times better than the HA configuration when considering the metabolic cost. In other words, taking advance of the work of Rafiq et al. (2017), it is possible to derive a simple linear equation from the following points: a walking speed of 4 km/h requires 2.9 MET. A walking speed of 1.7 km/h requires 2.3 MET. The equation is as follows: y = a *x+b. Where y is the MET and x is the speed, while a = 0.26 and b = 1.86. Therefore, if we substitute the metabolic cost found for the NOE and the HAA we can solve the equation for the hypothetical walking speed (x). The hypothetical walking speed of the NOE modality is 2.8 km/h, while the hypothetical walking speed for the HAA modality is 2 km/h. This underlines the fact that even an improvement of a few percentage points, such as is seen comparing HA to HAA, strongly impacts the reduction in metabolic consumption.

### 6.2. Discussion on the muscle fatigue analysis

The results obtained from the analysis of the MF do not confirm the study on metabolic consumption. In particular, the HA strategy does reduce the metabolic cost but increases muscle fatigue, while the HAA strategy reduces both the metabolic cost and muscle fatigue. This effect arises because, in HA, the lower part of the leg muscle involved in controlling the knee and ankle is required to work more. This happens mainly during the elongation of the elastic band. We see from Figure 6 that the tibialis and gastrocnemius activate more than the baseline. This is particularly evident for the gastrocnemius during the period of the gait cycle when the elastic tendon is elongating (from 5 to 55%).

On the other hand, for the tibialis, the compensation (higher muscular activation than baseline) occurs during the assistance phase (60−65%), thus showing a counter effect, which could be necessary to improve balance. These results do confirm that the HAA configuration performs better than the HA arrangement (Table 3). Indeed, only the HAA generates an effective advantage on the user’s energy balance.

The results confirm that the design of the assistive strategies must consider muscle synergies. When considering the HA configuration, where a single bilateral QPA is applied to the hip flexion, this assistive configuration is enough to counter the additional weight of the device and, at the same time, reduce metabolic expenditure. While the HAA configuration, which has two bilateral QPAs (hips flexion and ankles plantarflexion), performs better also, reducing muscle fatigue in three of the four assessed muscles.

### 6.3. Discussion on the muscle pattern analysis of the HA and HAA

The muscle activation of RF, VM, TA, and GL are evaluated during the walking task to quantify the energy exchange for both exosuit configurations. The results show that the control configuration strongly affects the muscle pattern during walking. Moreover, different control configurations, particularly this type of QPA, involve a high degree of human-robot interaction and flow of energy exchange, leading to diverse muscle activity trends, with more marked increases and decreases with respect to the baseline (Figures 6, 7).

The results show in both configurations (HA and HAA) the generation of different effects during distinct phases of the gait cycle due to the sequence of resistive and assistive phases. Therefore, the controller must be well-designed/tuned regarding actuation timing (Di Natali et al., 2020) and actuation configuration. When comparing the HA and HAA configurations, the HAA performs better than the HA in RF, VM, and GL by increasing the assistance and decreasing the resistance (Figures 6, 7). Focusing on the normalized and weighted RF activity for both the HA (assistance of 10%, resistance of 11%), and the HAA (assistance of 12.5%, resistance of 6.5%), show that a more biomimetic assistive configuration (i.e., HAA) enhance the assistive effects at the expenses of the resistive once. Thus, the HAA provides better performance than the HA configuration. Even though the result is slight, the HA affects the balance between the assistive and the resistive effects on the RF. This has unwanted impacts on the VM, TS, and GL, with considerable resistance effects. The side effects on the VM and the GL are strongly reduced by introducing assistance for the ankle plantarflexion (as in the HAA). In the HA, the normalized and weighted VM activity (assistance of 2.2%, resistance of 65.5%) and for the GL (assistance of 2.4%, resistance of 41.4) show an equilibrium shifted toward the resistive effect when compared with the HAA (VM assistance of 5.7%, resistance of 11.4%, and GL assistance of 10.7%, resistance of 9.5%). For the TA (mostly activated during ankle dorsiflexion and providing balance), we noticed that both configurations cause similar effects, with the resistance effect being more marked (assistance of about 9.5%, resistance of about 20%). We can assume that the walking pattern is modified for both configurations. Thus, unwanted effects and compensatory behaviors have to be counteracted.

This analysis demonstrates that when the actuation strategy is designed accordingly with muscle synergy, as in the HAA, efficiency increments due to augmentation of the assistance and a reduction in the resistive effects are more evident (particularly for the RF and the GL). On the other hand, the undesirable effects on the TA could lead to rethinking the actuation strategy and timing to reduce the side effects.

## 7. Conclusion and future works

This study compares two assistive strategies applied at hips and ankles employing QPAs integrated on the XoSoft exosuit, particularly vital as motions of the hip and ankle are considered important in assisting the elderly with walking and may ultimately be useful in reducing or even preventing falls. Hence, this work suggests that this exosuit may provide immediate improvements in walking performance by reducing muscle fatigue and metabolic expenditure. This makes this a very promising approach that could have essential benefits in activities of daily living for the elderly.

The investigation also evaluates the effective muscular reduction as a net balance between the storage and release phases for both exosuit configurations. Also importantly, side effects on the walking pattern and muscle activation due to the wearing of an external device are found to be strongly reduced on the VM when using the HAA configuration. At the same time with the TA, compensatory behaviors in both control configurations were found to be negligible. This analysis underlines the importance of a biomimetic assistive strategy to improve results and reduce compensatory actions.

Finally, by investigating how healthy subjects respond to the assistance provided by the QPA-powered exosuit, this study serves to define the technology’s potential for improving mobility and enabling future potential rehabilitation benefits. In fact, not only could the net assistance be used to enhance activities of daily living or rehabilitation, but it may also be possible to take advantage of the resistive phase of each QPA to train muscles in specific gait phases or generate established controlled compensatory effects. Future work will evaluate the impact on inexperienced subjects and the relevance of the learning curve.

The highly encouraging results from this study will drive further investigations in a broader population (particularly the elderly and those suffering from gait disorders with low level mobility impairments) and studies into compensatory behaviors that arise when assisted by different actuation strategies.

## Data availability statement

The raw data supporting the conclusions of this article will be made available by the authors, without undue reservation.

## Ethics statement

The studies involving human participants were reviewed and approved by the Ethical Committee of Liguria. The patients/participants provided their written informed consent to participate in this study. Written informed consent was obtained from the individual(s) for the publication of any potentially identifiable images or data included in this article.

## Author contributions

CD contributed to the conception and design of the study and conduction of the experimental section and contributed on the manuscript drafting. All authors contributed to manuscript revision, read, and approved the submitted version.
